# An Unusual Case of Perineural Infiltration and Orbital Invasion of Squamous Cell Carcinoma Associated with Actinic Keratosis

**DOI:** 10.1155/2020/8831668

**Published:** 2020-12-30

**Authors:** Edgard Farah, Marie Callet, Augustin Leclerc, Tryfon Rotsos, Chrysanthos Symeonidis, Pierre-Vincent Jacomet, Olivier Galatoire

**Affiliations:** Fondation Ophtalmologique Adolphe de Rotschild, 29 Rue Manin, 75019 Paris, France

## Abstract

Actinic keratosis is considered a precancerous lesion, constituting a precursor to squamous cell carcinoma (SCC) formation. Perineural invasion has been observed in patients with cutaneous carcinoma due to local subcutaneous tissue destruction and primarily involves the trigeminal nerve due to rich innervation provided by the supraorbital nerve in addition to the facial nerve. An unusual case of perineural infiltration and orbital invasion of squamous cell carcinoma associated with actinic keratosis is presented. A 70-year-old Caucasian woman presented with complete left eye ophthalmoplegia, total left upper-eyelid ptosis, and facial pain with paresthesia. Computed tomography revealed a process of the soft tissues in the left cheek infiltrating the infraorbital canal, pterygopalatine fossa, inferior orbital fissure, and left cavernous sinus with periosteal adherence. Magnetic resonance imaging revealed pathological extension via the left infraorbital canal with a considerable area of necrosis. Treatment of facial actinic keratosis may not prevent malignant transformation and can delay diagnosis and treatment of SCC. A deep biopsy appears to be essential for a correct diagnosis. Perineural spread of cutaneous SCC may be characterized by insidious progression in the cranial trigeminal nerve, abnormal ocular motility, diplopia, or external ophthalmoplegia.

## 1. Introduction

Actinic keratosis (AK) is considered a precancerous lesion, constituting a precursor to squamous cell carcinoma (SCC) formation [1]. The risk of evolving into an SCC increases with the intensity of exposure to UV radiation and with the use of immunosuppression while perineural invasion is observed in a small percentage of cases [2]. We present an unusual case of perineural infiltration and orbital invasion of squamous cell carcinoma associated with actinic keratosis.

## 2. Case Report

A 70-year-old Caucasian woman presented with complete left eye ophthalmoplegia, total left upper-eyelid ptosis, and facial pain with paresthesia in the dermatome of the first (V1) and second (V2) branches of the trigeminal nerve that was established gradually in the duration of the previous 6 months. She also complained about visual deterioration in the left eye.

**Figure 1:**
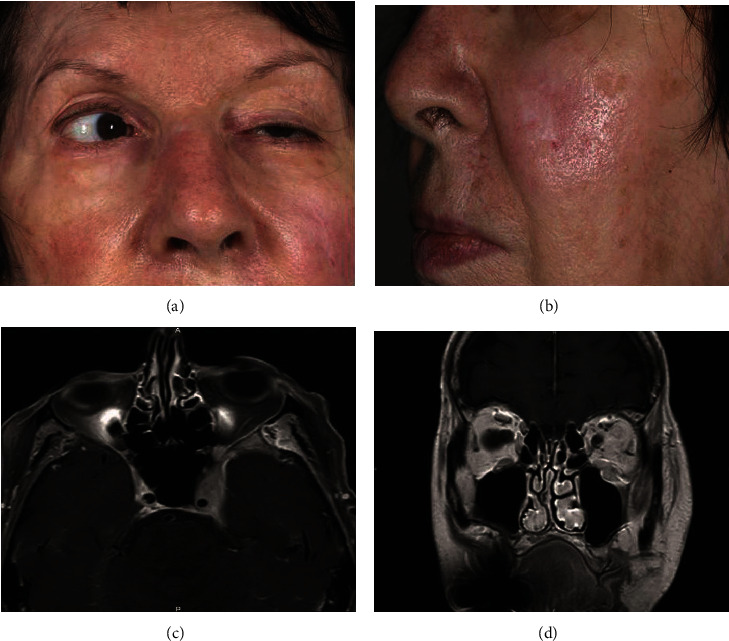
(a) Complete left ophthalmoplegia and left upper-eyelid ptosis. (b) Actinic keratosis of the left cheek. (c) Facial and orbital MRI: sagittal view: infiltrating lesion extending through the left infraorbital canal orbital apex and the left cavernous sinus with perineural infiltration. (d) Facial and orbital MRI: coronal view: infiltrating left orbital process started from the left cheek with a large amount of necrosis.

Regarding her medical history, she underwent photodynamic laser treatment two years prior to the baseline visit for a left cheek actinic keratosis (Figure 1(a)) and recently was prescribed an ingenol mebutate ointment with a satisfactory aesthetic skin result.

Clinical examination revealed complete left III, IV, and VI nerve palsy (Figure 1(b)) and trigeminal hypoesthesia with left face numbness. Best-corrected visual acuity (BCVA) in the left eye was 6/15 Snellen, and 6/60 in the right eye.

A computed tomography (CT) scan revealed an infiltrating process of the soft tissues in the left cheek infiltrating the infraorbital canal, the pterygopalatine fossa, the inferior orbital fissure, and the left cavernous sinus with a periosteal adherence. Magnetic resonance imaging (MRI) revealed pathological extension via the left infraorbital canal of the 30 × 27 mm diameter soft tissue jugal process with a considerable area of necrosis. It was located in the fat of the anterior area of the left maxillary sinus and was infiltrating toward the deep facial massif. A second homogenous infiltrating process in the deep facial massif was described, invading the left masticator space, the pterygopalatine fossa, the temporal anterior left meninx, the orbital apex, and the left cavernous sinus with perineural infiltration (Figures 1(c) and 1(d)).

A punch biopsy of the left cheek was performed. *Η*istology revealed actinic keratosis with diffuse nonspecific inflammatory and fibrotic lesions located in the epidermis and the dermis. The patient underwent a later deep orbital floor biopsy with an infraorbital trigeminal V2 nerve biopsy under general anesthesia. The histopathologic examination demonstrated a poorly differentiated SCC invading the infraorbital nerve and the periosteum with perineural involvement. Histological markers for EMA, AE1, and AE3 were positive.

Positron emission tomography (PET)/computed tomography (CT) did not reveal any primary tumor of secondary metastatic localization but micronodular parenchymatous pulmonary lesions of the mid and inferior right lung. Subsequently, the patient underwent chemotherapy with paclitaxel/fosfamid/cisplatin, and 4 1-week cycles of 70 Gy wide-field radiation therapy of the left cheek and cervical area were performed. At the time, clinical examination revealed no signs of recurrence of the cheek SCC and on follow-up CT scans. The symptoms remained unchanged with a complete left eye ophtalmoplegia, altered sensation, and intermittent acute pain in the left trigeminal area.

## 3. Discussion

In the context of SCC, perineural invasion has been observed in 3-14% of patients with cutaneous carcinoma due to local subcutaneous tissue destruction [3]. Perineural invasion predominantly involves the trigeminal nerve due to rich innervation provided by the supraorbital nerve in addition to the facial nerve [4, 5]. The most common presentation is decreased sensation in the area innervated by the trigeminal nerve and caused by an SCC located in the forehead or brow [6].

According to Feasel et al., 60-70% of the patients with perineural invasion are asymptomatic in the early stages of the disease. Perineural invasion is not considered until orbital and cavernous sinus involvement is observed and implies an often delayed diagnosis [7]. Consequently, the prognosis remains poor and treatment limited as radical surgery is often impossible due to cavernous sinus and facial nerve branch involvement [8–10].

Local treatment of facial actinic keratosis may not prevent malignant transformation and can delay diagnosis and treatment of SCC, often diagnosed at a late stage. A deep biopsy appears to be essential for a correct diagnosis. Abnormal ocular motility, diplopia, and external ophthalmoplegia must be considered a red flag for perineural spread of cutaneous squamous cell carcinoma with insidious progression in the cranial trigeminal nerve.

## Data Availability

Relevant data is available on request. Please contact Dr. Chrysanthos Symeonidis (chrys2209@gmail.com).
